# The Predictive Coding Account of Psychosis

**DOI:** 10.1016/j.biopsych.2018.05.015

**Published:** 2018-11-01

**Authors:** Philipp Sterzer, Rick A. Adams, Paul Fletcher, Chris Frith, Stephen M. Lawrie, Lars Muckli, Predrag Petrovic, Peter Uhlhaas, Martin Voss, Philip R. Corlett

**Affiliations:** a Department of Psychiatry, Campus Charité Mitte, Charité – Universitätsmedizin Berlin, Berlin, Germany; b Department of Psychiatry and Psychotherapy, Charité University Medicine and St. Hedwig Hospital, Berlin Center for Advanced Neuroimaging, Humboldt University Berlin, Berlin, Germany; c Division of Psychiatry, University College London, London, United Kingdom; d Wellcome Trust Centre for Neuroimaging, University College London, London, United Kingdom; e Department of Psychiatry, Addenbrooke’s Hospital, University of Cambridge, Cambridge, United Kingdom; f Wellcome-MRC Behavioral and Clinical Neuroscience Institute, Cambridge and Peterborough Foundation Trust, Cambridge, United Kingdom; g Center for Clinical and Brain Sciences, Division of Psychiatry, Royal Edinburgh Hospital, University of Edinburgh, Edinburgh, United Kingdom; h Centre for Cognitive Neuroimaging, Institute of Neuroscience & Psychology, College of Medical, Veterinary and Life Sciences, University of Glasgow, Glasgow, United Kingdom; i Department of Clinical Neuroscience, Karolinska Institutet, Stockholm, Sweden; j Department of Psychiatry, Yale University, New Haven, Connecticut

**Keywords:** Bayesian brain, Cognition, Delusions, Hallucinations, Learning, Perception, Predictive coding, Schizophrenia

## Abstract

Fueled by developments in computational neuroscience, there has been increasing interest in the underlying neurocomputational mechanisms of psychosis. One successful approach involves predictive coding and Bayesian inference. Here, inferences regarding the current state of the world are made by combining prior beliefs with incoming sensory signals. Mismatches between prior beliefs and incoming signals constitute prediction errors that drive new learning. Psychosis has been suggested to result from a decreased precision in the encoding of prior beliefs relative to the sensory data, thereby garnering maladaptive inferences. Here, we review the current evidence for aberrant predictive coding and discuss challenges for this canonical predictive coding account of psychosis. For example, hallucinations and delusions may relate to distinct alterations in predictive coding, despite their common co-occurrence. More broadly, some studies implicate weakened prior beliefs in psychosis, and others find stronger priors. These challenges might be answered with a more nuanced view of predictive coding. Different priors may be specified for different sensory modalities and their integration, and deficits in each modality need not be uniform. Furthermore, hierarchical organization may be critical. Altered processes at lower levels of a hierarchy need not be linearly related to processes at higher levels (and vice versa). Finally, canonical theories do not highlight active inference—the process through which the effects of our actions on our sensations are anticipated and minimized. It is possible that conflicting findings might be reconciled by considering these complexities, portending a framework for psychosis more equipped to deal with its many manifestations.

There is a pressing need to understand and better treat psychosis (i.e., psychotic symptoms and psychotic disorders). While dopamine antagonists are effective, many patients experience residual symptoms (1). They have poor functional outcome and a high risk of suicide (2). Furthermore, the side effects of many antipsychotics can lead to poor adherence. Here, we argue that single-level accounts of psychosis, such as the dopamine hypothesis, are too reductionist on their own and will achieve full value only when embedded in a more complex explanatory framework that unites several levels of explanation [e.g., Maia and Frank (3)]. Predictive coding and Bayesian inference 4, 5, 6 may provide such a framework, linking the neurobiology of psychosis with its clinical phenomenology by way of computational processes. We will critically evaluate this framework and suggest future lines of inquiry.

## Predictive Coding as Hierarchical Bayesian Inference

**Figure 1:**
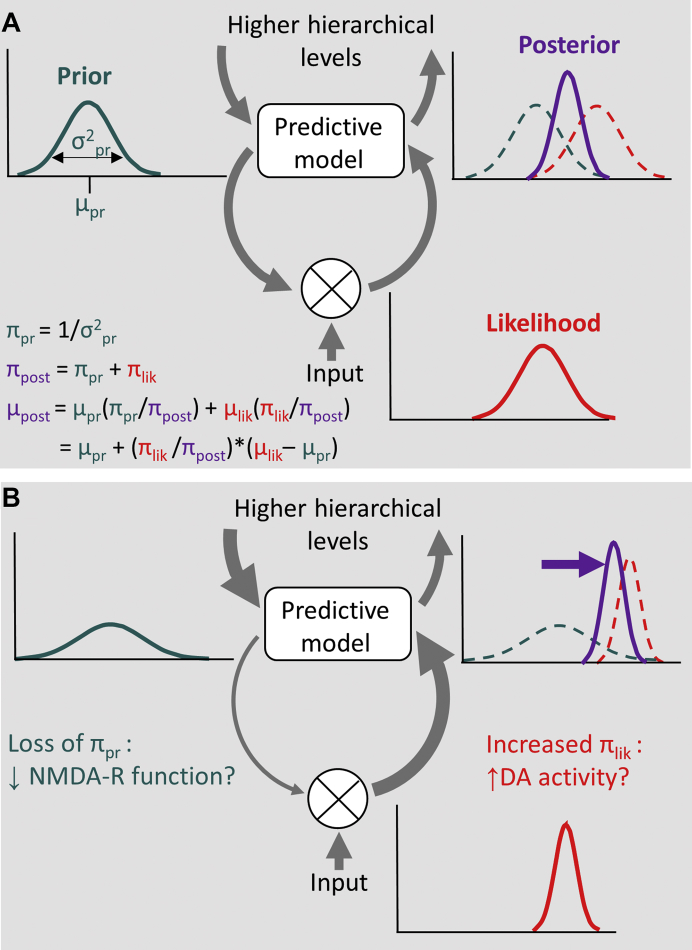
Schematic illustration of Bayesian predictive coding as an explanatory framework for psychosis. **(A)** Predictions are encoded at higher levels of a hierarchical system and are sent as predictive signals to lower levels (downward arrows on the left). Whenever the incoming sensory data violate these predictions, a prediction error signal is sent to update the predictive model at higher levels (upward arrow on the right). Formalized as Bayesian inference, predictions (prior) and sensory data (likelihood) are represented in the form of probability distributions. The posterior results from the combination of prior and likelihood according to Bayes’ rule, weighted by their respective precisions π (which is the inverse of their variance σ; see first equation), and updates the predictive model (third equation). The fourth equation rearranges the third to show that the new posterior mean is simply the old prior mean added to a precision-weighted prediction error. **(B)** In psychosis, the balance between predictions and sensory data has been proposed to be disrupted, with a decreased precision in the representation of priors and increased precision of the likelihood (59). This imbalance biases Bayesian inference toward the likelihood and away from the prior, resulting in the abnormally strong weighting of prediction error. Candidate mechanisms for decreased prior and increased likelihood precisions are hypofunction of glutamatergic *N*-methyl-D-aspartate receptors (NMDA-Rs) and increased dopamine (DA) activity, respectively. Some psychotic phenomena may be explained by a compensatory increase in feedback signaling at higher levels of the hierarchy (bold arrow, upper left).

Von Helmholtz’s (7) idea of unconscious inference held that the brain uses learned predictions to infer the causes of incoming sensory data. This process can be formalized as Bayesian inference 5, 8, whereby a probabilistic prediction (prior) is combined with observed sensory data (likelihood) to compute a posterior probability (posterior). The posterior corresponds to the percept that is most likely, given the prior and the likelihood (9). This may be implemented in the brain through predictive coding, but there are alternatives 10, 11. Predictive coding conceives of the brain as a hierarchy whose goal is to maximize the evidence for its model of the world by comparing prior beliefs with sensory data, and using the resultant prediction errors (PEs) to update the model (Figure 1). Model evidence can also be maximized through active inference—that is, by acting on the world (and thus selecting sensory evidence) to minimize PEs (12). Moreover, hierarchical Bayesian inference entails modeling ourselves as agents who change the world: indeed, in this scheme, experiences such as agency and selfhood are inferred from the consequences of our own actions (13).

In terms of neural implementation 14, 15, predictive signals may be sent from higher hierarchical levels predominantly via glutamatergic *N*-methyl-D-aspartate receptor (NMDAR) signaling; any disparity between prior belief and sensory data is then signaled as a PE to the higher levels, mostly via glutamatergic alpha-amino-3-hydroxy-5-methyl-4-isoxazole propionic acid receptors. Animal and human studies of vision support this hypothesis 16, 17, 18, 19. In Bayesian terms, the PE corresponds to the difference between the means of the prior and the likelihood distributions and is weighted by their relative precisions (20), whereby precision corresponds to the inverse variances of their respective probability distributions (Figure 1A). Roughly, this can be thought of as the relative reliability of priors or sensory data, the extent to which each colors current inference and learning by weighting the impact of PEs (20). Precision is thought to be signaled by neuromodulators such as dopamine and acetylcholine, depending on the particular inferential hierarchy 21, 22, 23. Perturbations in these neuromodulators are thus candidates for the profound departures from consensual reality that characterize psychotic states (24).

Functional magnetic resonance imaging has shown that feedback from higher- to lower-level sensory cortices carries spatiotemporally precise and context-specific predictions 25, 26, 27, 28, 29. When predictions are confirmed by sensory input, this leads to a dampening of neural responses 30, 31, while violation of predictions leads to enhanced responses compatible with PE signaling (26). Electrophysiological studies investigating neural responses to deviant stimuli, such as the mismatch negativity, suggest a hierarchical organization of prediction and PE signaling 32, 33, 34. In the time-frequency domain, oscillatory signals have been related to predictive coding, with feedback signaling of predictions being mediated predominantly by the alpha/beta frequency bands and feedforward PE signaling by gamma-band activity 14, 35, 36, 37.

There is a deep relevance of this account to psychosis, in terms of both neurobiology (glutamatergic and dopaminergic systems in schizophrenia, acetylcholine in hallucinosis) and phenomenology (perception, beliefs, agency, and ipseity). We now outline previous theories of psychosis that are highly relevant to the predictive coding account (38).

## Precursor Theories of Predictive Coding

Anticipating the focus of predictive coding accounts on perceptual inference, and in line with phenomenological observations 39, 40, 41, early theories of psychosis emphasized altered perception. Maher (42) highlighted the failure to integrate sensory input with learned expectations, which was further developed by Gray *et al.* (43) and Hemsley and Garety (44). Hemsley and Garety (44) put forth the first explicitly Bayesian analysis of delusions, suggesting how belief, evidence, and their disrupted interaction could garner aberrant inference. Hemsley and Garety (44) and Gray *et al.* (43) argued that perception proceeded through modeling of the world and that neural signals normally evoked by surprising events are inappropriately engaged in psychosis. As a consequence, patients attend to and learn about events that others would ignore, forming the grounds for both hallucinations and delusions. A similar idea was later developed in the wake of fundamental discoveries regarding the role of dopamine in motivational salience and reward PE signaling 45, 46. Heinz (47) and Kapur (48) proposed that excessive dopamine signaling results in a misattribution of salience to normally inconspicuous events, which then demand explanation, culminating in delusions. Another influential theory of psychosis, the comparator model, suggested impaired predictive signaling as a key mechanism underlying hallucinations (49) and later so-called passivity phenomena, such as the experience of one’s actions or thoughts being externally controlled (50). The comparator model proposes a failure to predict one’s own actions owing to impaired corollary discharge, which normally serves to predict and explain away the sensory consequences of self-initiated actions. Later versions suggested that the consequences of any action are predicted by a neural forward model (51) and that it is the reduced precision of these predictions that leads to the experience of alien control 13, 52.

Most of these models focused on one specific symptom dimension. However, the above-chance co-occurrence of a number of characteristic phenomena in psychotic disorders demands theories that can accommodate multiple symptoms. Moreover, most earlier theories failed to integrate the multitude of documented neurobiological abnormalities and focused on one particular mechanism while disregarding others. For instance, while the idea of salience misattribution related delusions primarily to dopamine dysfunction, more recent accounts along these lines have provided a broader picture by outlining how dopaminergic dysfunction may be linked to altered glutamatergic and gamma-aminobutyric acidergic neurotransmission 53, 54. Meanwhile, neurocognitive theories have made advances largely at the conceptual level. Empirical tests of these theories could yield evidence for a theory or against it but could not provide quantitative, mechanistic evidence. Predictive coding can provide such mechanistic evidence by estimating model parameters at the level of the individual 55, 56, 57, and relating those parameters to the severity and type of psychotic symptoms.

## A Predictive Coding Account of Psychosis

In Bayesian predictive coding schemes, the PE is affected by the precision of the sensory data: if it is high, the precision-weighted PE in case of a mismatch will be greater, and vice versa (Figure 1A)—just as in classical statistical inference, the *t* statistic is greater if the standard error of the data is smaller. Furthermore, the degree to which a prior belief will change in response to a PE is also determined by its own precision: an imprecise prior will update more than a precise one will. It is crucial to represent accurately the precisions of both prior beliefs and sensory data, as a failure to do so will lead to false inferences (just as overestimating the precision of the data causes type I errors). Psychosis has been related to a decreased precision of prior beliefs and/or increased precision of sensory data 13, 24, 58, 59, 60, 61. This imbalance in precisions shifts the posterior toward the sensory data and away from the prior (Figure 1B), and inference is thus driven more strongly by the sensory data.

This notion, which we here refer to as canonical predictive coding account of psychosis, is supported by several lines of evidence. For example, psychosis has been associated with a greater resistance to visual illusions (which rely on prior beliefs for their effects), a failure to attenuate sensory consequences of self-generated actions, impaired smooth visual pursuit of a moving target, but improved tracking of unpredictable changes in target motion, a decreased influence of stimulus predictability on brain responses [e.g., N400, P300, mismatch negativity; but see Erickson *et al.* (62)], and a loss of corticothalamic connectivity [for reviews, see Adams *et al.* (59) and Notredame *et al.* (61)]. The main neurotransmitter alterations that are thought to underlie this predictive coding abnormality are hypofunction of cortical NMDARs and gamma-aminobutyric acidergic neurons as well as elevated striatal dopamine D_2_ receptor activity, as reviewed elsewhere 24, 59, 63. The resulting aberrant encoding of precision could lead to an abnormally strong weighting of PEs, which in turn leads to aberrant learning and the formation of delusional beliefs 53, 58, 59, 64. This canonical predictive coding account of psychosis is not without controversy. Some frank psychotic symptoms have been related to increased prior precision and therefore a stronger impact of prior beliefs. We return to this issue below.

One strength of predictive coding is that it is more generalized than earlier accounts, which tended to localize the pathology to a specific brain area or psychological function, e.g., the pathway connecting the subiculum to the nucleus accumbens (43), striatal dopamine release 47, 48, or altered corollary discharge (50). By providing a generic framework compatible with previous neurocognitive theories and neurobiological data, predictive coding also holds promise of accounting for more than one psychotic symptom. It provides a plausible explanation not only for delusional mood and paranoid delusions, akin to the aberrant salience account 47, 48, but also for hallucinations 61, 63 and passivity phenomena 13, 59. On the predictive coding view, corollary discharge becomes a prediction of the sensory consequences of action. A failure of that prediction renders those consequences surprising, garnering the inference that actions were under external control rather than self-authored.

While predictive coding thus has the potential to unify accounts of psychosis (59) and integrate empirical evidence at different levels of observation and within a formal quantitative model, a number of important challenges remain.

### The Heterogeneity of Psychosis

The heterogeneity of psychosis and the fact that delusions and hallucinations co-occur, but to varying degrees, demands explanation. However, an overly flexible or general theory that explains everything will be of little use. In our view, predictive coding puts forward a skeletal understanding of how, given a perturbation to a component of the model, the phenomenological outcome has particular characteristics. In other words, predictive coding does not reduce psychosis to a single cause, but rather attempts to show how different underlying pathophysiologies could perturb the system in ways that produce overlapping phenomenologies.

This challenge is exemplified by arguments as to whether a single deficit within a predictive coding model can explain both perceptual and cognitive aspects of psychotic symptoms. The two-factor account (65) invokes both perceptual and cognitive problems in the genesis of some delusions, based on the observation of both abnormal percepts and bizarre explanations of these percepts. According to predictive coding, reduced precision of priors could potentially account for both factors, given that it would alter perceptual inference and make cognitive explanations for altered percepts less constrained (58). Recent neurobiological work, however, has raised the question of whether a loss of prior precision (e.g., prefrontal hypoconnectivity) and gain in sensory precision (e.g., sensory hyperconnectivity) may indeed be two separate factors in the illness 66, 67. These observations might be reconciled by adding some nuance to the single-layer predictive coding example outlined above. Predictive coding actually takes place across large multilevel hierarchies in which the precision weighting of PEs may be controlled—at least in part—independently at different levels and in different sensory modalities (68). Thus, NMDAR (or other neuromodulatory) dysfunction may have widespread and diverse effects on the precision of prior beliefs in perceptual and cognitive domains. Furthermore, NMDAR-mediated interneuron dysfunction may not only disinhibit (i.e., amplify) sensory areas, but also reduce the stability of more sustained representations in higher areas (i.e., reduce the signal-to-noise ratio), leading to increased sensory and decreased prior precision, respectively.

A recent study emphasized the importance of analyzing the different weightings of priors that may be implemented at different hierarchical levels. The authors probed the use of prior knowledge to perceive the gist versus the details of ambiguous images in a healthy population with varying degrees of hallucination and delusion proneness (69). Hallucination proneness correlated with stronger employment of global (gist) and local (detail) priors, whereas delusion proneness was associated with less reliance on local priors. This raises a hitherto underappreciated mechanism through which the heterogeneity in psychotic phenomenology could be explained, namely differential weightings of specific hierarchical levels in different psychotic symptoms (70). The neural circuits and neurochemical mechanisms of these effects ought to be established. Where to draw the line between perceptual and conceptual processing remains a challenge, and indeed, whether and how high-level prior beliefs modulate perceptual processes is controversial (71). However, recent neural data suggest that they do 72, 73, and that the impact of priors on perception may be enhanced in those with hallucinations 74, 75, 76.

### Hallucinations: Strong or Weak Priors, or Both?

Hallucinations represent a challenge, as two apparently opposing aberrations have been proposed and there is evidence supporting both. One view has linked hallucinations to a failure to attenuate sensory precision, including the sensory consequences of inner speech, analogous to the mechanism that is thought to underlie delusions of control 58, 77, 78, 79, 80. This would correspond to the notion of low precision of priors relative to a disproportionately high precision of neural signals that encode inner speech in auditory cortex, akin to the canonical predictive coding account. Indeed, hallucination severity in patients with schizophrenia is associated with a failure to attenuate predictable signals in the somatosensory cortex (81). Similarly, a model-based functional magnetic resonance imaging study using probabilistic presentation of speech stimuli found diminished auditory cortex PE-related activations and deactivations to the unexpected presence or absence of speech, respectively, in patients with hallucinations, suggesting aberrant PE signaling (82).

Alternatively, hallucinations may result from enhanced rather than weakened top-down predictive signaling (i.e., increased precision of priors) on neural activity in sensory cortices (83). Perception would therefore rely less on the sensory input and more on beliefs. Supporting this notion, directional bottom-up connectivity from Wernicke’s to Broca’s areas is reduced in individuals who hear voices (84). Top-down predictions from Broca’s area may thus be less constrained by sensory information. Recently, people who hear voices were found to be more susceptible to conditioning-induced hallucinations, and accordingly, modeling in a Bayesian framework showed stronger perceptual priors (74). Another recent study investigated the perception of auditory stimuli under different levels of uncertainty (75). Hallucinations in schizophrenia patients correlated with a perceptual bias that reflected increased weighting of prior beliefs. This bias could be pharmacologically induced by amphetamine and strongly correlated with striatal dopamine release. Together, these findings favor a strong-prior account of hallucinations and thus call into question the suggestion that aberrant salience of inner speech confers the content of voices.

How can these apparently contradictory findings be reconciled? The auditory system may have a strong prior for speech—perhaps because this is a highly salient signal for our species—and as such, noisy signals in the auditory cortex are resolved by that prior into perceived speech (akin to our propensity to see faces in clouds, for example). At the same time, corollary discharge (i.e., descending predictions regarding the consequences of action) may still have a role, in ascribing agency to those experiences. In this case, disruption of corollary discharge as a form of predictive signaling may be more broadly relevant for both hallucinations and delusions, which entail aberrant inferences about both agency and the intentions of others. This may explain the lack of specificity of corollary discharge deficits to specific positive symptoms 85, 86.

Furthermore, priors at low and high hierarchical levels may be differentially affected. Neurobiologically, this may be mediated by the higher density of recurrent connections in higher-level association cortices, compared with primary sensory regions, such that a psychotogenic perturbation that impacts excitatory/inhibitory (E/I) balance may have more profound effects higher rather than lower in the hierarchy (87) [see Jardri and Deneve (10) for a detailed exposition of the role of E/I balance in learning, inference, and psychosis]. In brief, the E/I relationships may implement exactly the predictive cancellation mechanisms that underlie predictive coding. Blocking NMDARs (with ketamine for example) profoundly alters E/I balance 88, 89, thus altering the balance between priors and PEs (24), perhaps differently at different hierarchical levels (87). Many findings in psychotic or psychosis-prone individuals point to weak priors that are implemented at low levels [e.g., visual illusions; see above and 24, 59, 63]. Impaired predictive coding at low levels may result in perceptual uncertainties that may be (partly) compensated by reliance on high-level abstract or semantic prior beliefs (Figure 1B). This may result in a top-down enhancement of signals in sensory cortices, thus facilitating hallucinations. There are even data suggesting that psychotic individuals with and without hallucinations utilize different priors to different extents in the same task. Powers *et al.* (74) found that people with hallucinations had strong perceptual priors that were not present in psychotic patients who did not hallucinate and who, indeed, may have had weak priors. The presence of strong priors and their immunity to updating were associated with strong insula and hippocampal responses, respectively (74). These psychological and circuit observations should be replicated, manipulated with transcranial magnetic stimulation (90) or real-time neurofeedback (91), and the mediating role of glutamate and E/I balance at different hierarchical levels should be explored in human pharmacological and patient studies as well as animal models.

### Changes in Psychotic Phenomenology Over Time

Another important challenge for theories of psychosis is that the pathophysiology may change over the course of the underlying disorder (92). While changes of symptomatology over time were emphasized by phenomenologists 93, 94, they are largely neglected by current classification systems. For example, delusions are often highly fixed and incorrigible in chronic patients, while they are still malleable in early psychosis (24). With time and treatment, they may become less impactful on function. Thus, the underlying pathophysiology may also change over time and differentially contribute to psychopathology at different stages of illness. Evidence from magnetic resonance spectroscopy suggests that alterations in glutamatergic neurotransmission may change over the course of schizophrenia 95, 96. Indeed, ketamine infusion in healthy volunteers may better mimic the E/I dysbalance and hierarchical perturbations observed in first-episode patients than in those with more chronic illness (97). We note with interest that the metabotropic glutamate agonist pomaglumetad appears to have efficacy in early rather than chronic schizophrenia, suggesting that hyperglutamatergia is more involved around the onset and early phases of illness 98, 99. The issue is further complicated by the possibility that such changes over time are not limited to aspects of brain development and learning, but rather involve ongoing neurobiological and environmental influences, including effects of antipsychotic medication and drug use. Current data are consistent with the idea that with chronicity, prefrontal glutamate signaling may progress from an excess to an insufficiency. Future work with magnetic resonance spectroscopy and electrophysiological markers of E/I balance could track these changes and pinpoint their effects on predictive coding (100). More broadly, in predictive coding, the brain is involved in a dynamic prediction-based negotiation with the world, which evolves as the person tries out new models of reality. While they eventually settle on beliefs that become engrained, one would expect the patient's priors to evolve across time.

### The Persistence of Psychotic Experiences

An important unresolved question is how aberrant predictive coding might account not only for the emergence of delusions, but also for their persistence. It is a defining feature of delusions that they persist despite contradicting evidence. This suggests an excessive influence of delusional beliefs on the perception of new information [e.g., (101)], which would entail an increased precision of delusion-related priors. In contrast, the emergence of delusions might result from decreased precision of priors as outlined above 24, 58, 59 (Figure 1B). Evidence from experiments using the NMDAR antagonist ketamine, which has been previously shown to induce aberrant PEs, suggests a link between PE signaling and memory reconsolidation, which could strengthen delusional beliefs and foster their persistence 102, 103. An additional (or complementary) mechanism could be related to an imbalance between priors at low and high levels of the predictive coding hierarchy, as suggested by a series of studies investigating perceptual inference in relation to delusions 72, 104, 105, 106. In contrast to weak low-level priors, the effects of more abstract high-level priors may be abnormally strong (Figure 1B). Such a mechanism could sculpt perception into conformity with delusional beliefs and thus contribute to their persistence. An increased influence of learned high-level beliefs in relation to psychotic symptoms was also reported for the perception of images with impoverished sensory information where perceptual inference relies strongly on priors (107). Differential roles of priors at low and high levels of the hierarchy are also suggested by recent evidence relating delusion proneness to reduced usage of prior beliefs in perceptual but not cognitive decision making (108).

Furthermore, aberrant predictive coding could render other people unreliable, to be treated with suspicion. This could account for the social content of psychotic symptoms, but may also explain why they persist, and even strengthen, in the face of efforts to refute them (109). Perceptible social cues may be more uncertain than nonsocial ones, because they may or may not serve as reliable signals of others’ intentions, which we can never fully know (110). Consequently, high-level social priors may be particularly influential in the perception and beliefs of those with psychotic symptoms (109). There may also be a motivated quality to psychotic inferences (111). That is, psychotic symptoms may provide a form of personal identity, and personal-level data may be assumed to be more reliable than those from others. Finally, beliefs have value in and of themselves. Psychotic symptoms may be seen as attempts to garner some advantage, perhaps by convincing others of their veracity (111). This renders them susceptible to the same biases and asymmetries in updating observed with nondelusional beliefs (111). These asymmetries can be explained with a Bayesian model, if we allow agents to derive utility from their beliefs (112).

## A Roadmap for Future Research

Predictive coding was not conceived to explain psychosis. It is a general theory of brain function. If it is a useful theory of how the brain works, then it should also be useful to account for states of aberrant brain function such as psychosis. The question is therefore not whether there is one specific abnormality in predictive coding that can explain psychosis, but rather whether predictive coding provides a framework that can help us to better understand psychosis. We believe that its greatest strength is that it can be formulated in computational terms and therefore lends itself to rigorous quantitative testing. However, while there is abundant empirical evidence compatible with a predictive coding account, more research is needed that explicitly tests (and potentially falsifies) predictions derived from this theory. We therefore advocate research that addresses the outlined challenges head-on, in a hypothesis-driven way, and with the methodological rigor that is provided by the computational framework.

One key question that has received too little attention relates to the hierarchical nature of predictive coding. Potentially different roles of high and low levels of the hierarchy were highlighted throughout our discussion of important challenges to predictive coding. Such differences may resolve apparent inconsistencies regarding weak versus strong priors, help to understand the heterogeneity in the phenomenology of psychosis, and explain changes in symptomatology over time. Table 1 summarizes the theory and controversy regarding the predictive coding alterations underlying hallucinations and delusions. Experimental tasks are needed that reliably pinpoint predictive coding at low versus high levels of the hierarchy. Such tasks could then be used in conjunction with computational modeling [for a recent example, see Weilnhammer *et al.* (100)] to directly test, e.g., the hypothesis that delusions are related to weak low-level priors and hallucinations are related to strong high-level priors.

**Table 1:** Predictive Coding and Positive Symptoms: Theory and Controversy

Symptom	Feature	Theory	Literature	Controversy
Hallucinations	Percepts without external stimulus	Strong perceptual priors	Powers *et al.*(120)	Entails weak and strong prior beliefs—for perception and action—in the same brain at the same time
	Speech from external agents	Weak corollary discharge	Thakkar *et al.*(86)	
Delusions	Delusional mood/aberrant salience	Weak perceptual priors	Corlett *et al.*(121)	Necessitates a transition from weak to strong priors as delusions form, foment, and become ingrained
	Fixed in the face of contradictory evidence	Strong memory reconsolidation/strong conceptual priors	Corlett *et al.*(103); Schmack *et al.*(72)	

Here we highlight the facets of hallucinations and delusions that have been addressed by predictive coding–based theories. Each has garnered empirical support; however, overarching theories—grounded in a broader multisensory and enactive framework that can accommodate the evolution and trajectories of positive symptoms—are required. We focus here on hallucinations and delusions. For consideration of other psychotic symptoms such as thought disorder and passivity phenomena from the viewpoint of predictive coding, please see Griffin and Fletcher (109) and Sterzer *et al.*(13).

Another important direction will be research into neural markers of hierarchical feedback and feedforward processing and their relation to the precisions of prior beliefs and PEs, respectively, in Bayesian inference. Recent advances in the neuroimaging of laminar anatomical projection patterns will help in this regard 29, 36. Computational modeling should be used to examine how precision is reflected in neural measurements, and rigorous state-of-the-art model comparison is needed to probe predictive coding against other models of message passing. Pharmacological models are a promising approach to probe the roles of candidate neurotransmitter systems. Their direct comparison with neural predictive coding alterations in relation to specific psychotic symptom dimensions will help to address key challenges in psychosis research, such as the phenomenological heterogeneity of psychosis. Animal models should be further developed into an additional pillar of psychosis research, as important insights are expected from a more targeted manipulation of specific brain circuits and transmitter systems. For example, optogenetic manipulation of E/I balance (113) could be used to explore the computations underlying predictive coding. Similarly, models that identify how genes relate to brain development (114) and changes the canonical microcircuits (14) involved in aberrant predictive coding are warranted. Ideally, different levels of investigation should be translationally integrated within a common computational modeling framework.

At the level of symptoms, we need a better understanding of the processes underlying specific psychotic symptoms and their interrelationships. For example, delusion- and hallucination-related processes should be investigated at the same time in the same patients to examine how these neural and symptom processes are organized. Intriguing epidemiological data suggest a hierarchy from hallucinations to delusions (115). Indeed, what we learn about these processes should be applied at the level of diagnostic entities, with a number of possible implications. First, understanding the predictive coding mechanisms underlying psychosis may lead to the delineation of new entities within and across existing diagnostic groups such as schizophrenia and bipolar disorder. Second, models are needed that distinguish psychosis from other psychiatric syndromes. For example, current models of autism are strikingly similar to the predictive coding account of psychosis 116, 117, 118. Future investigations should examine differences and commonalities in neural computations in individuals with psychosis and those with autism (117). Third, predictive coding can relate psychosis to “normal” brain function, which may help to destigmatize the disorder (92): psychosis may be understood as a variety of brain function, in line with the so-called continuum view (119), which considers psychotic symptoms as extreme expressions of normal traits. “False inferences” made by psychotic individuals may be rendered comprehensible given the premises of predictive coding. As Adams *et al.* (59) stated, “From the point of view of the subject its inferences are Bayes-optimal. It is only our attribution of the inference as false that gives it an illusory or delusionary aspect.”

Importantly, a more complete model of psychosis may help patients understand their experiences, which could aid the development of psychotherapies. Moreover, predictive coding offers the possibility of more specific and quantitative predictions about symptoms and their mechanisms. Such an approach may in turn help not only to use those drugs that are currently available in a more targeted way, but also to develop new pharmacological interventions.
